# Effectiveness of an Online Programme to Tackle Individual’s Meat Intake through SElf-regulation (OPTIMISE): A randomised controlled trial

**DOI:** 10.1007/s00394-022-02828-9

**Published:** 2022-03-04

**Authors:** Kerstin Frie, Cristina Stewart, Carmen Piernas, Brian Cook, Susan A. Jebb

**Affiliations:** Nuffield Department of Primary Care Health Sciences, University of Oxford, Radcliffe Observatory Quarter, Woodstock Road, Oxford, OX2 6GG UK

**Keywords:** Self-regulation, Self-monitoring, Goal-setting, Meat intake, Meat reduction, Multi-component intervention

## Abstract

**Purpose:**

A reduction in meat intake is recommended to meet health and environmental sustainability goals. This study aimed to evaluate the effectiveness of an online self-regulation intervention to reduce meat consumption.

**Methods:**

One hundred and fifty one adult meat eaters were randomised 1:1 to a multi-component self-regulation intervention or an information-only control. The study lasted 9 weeks (1-week self-monitoring; 4-week active intervention; and 4-week maintenance phase). The intervention included goal-setting, self-monitoring, action-planning, and health and environmental feedback. Meat intake was estimated through daily questionnaires in weeks 1, 5 and 9. The primary outcome was change in meat consumption from baseline to five weeks. Secondary outcomes included change from baseline to nine weeks and change in red and processed meat intake. We used linear regression models to assess the effectiveness of all the above outcomes.

**Results:**

Across the whole sample, meat intake was 226 g/day at baseline, 118 g/day at five weeks, and 114 g/day at nine weeks. At five weeks, the intervention led to a 40 g/day (95%CI − 11.6,− 67.5, *P* = 0.006) reduction in meat intake, including a 35 g/day (95%CI − 7.7, − 61.7, *P* = 0.012) reduction in red and processed meat, relative to control. There were no significant differences in meat reduction after the four-week maintenance phase (− 12 g/day intervention vs control, 95% CI 19.1, − 43.4, *P* = 0.443). Participants said the intervention was informative and eye-opening.

**Conclusion:**

The intervention was popular among participants and helped achieve initial reductions in meat intake, but the longer-term reductions did not exceed control.

**Trial registration:**

ClinicalTrials.gov NCT04961216, 14th July 2021, retrospectively registered.

**Supplementary Information:**

The online version contains supplementary material available at 10.1007/s00394-022-02828-9.

## Background

Many motivations exist for individuals to reduce or stop their meat consumption, such as concerns about the environment, personal health, or animal welfare, as well as taste preferences [1]. However, it is research highlighting the negative impact of meat production on the environment and of meat consumption on human health that has led to calls for a shift towards more plant-based diets [2, 3]. In the UK, meat intake has decreased by 17 g/capita/day between 2008/09 and 2018/19, a reduction of 17% [4], but the National Food Strategy has recommended a reduction of 30% will be needed over the next decade to meet dietary targets for health and the environment [5].

Several barriers can hinder an individual’s ability to reduce their meat intake. First, the impact of meat consumption on both health and the environment is often underestimated [6], and providing this information has been found to boost intentions to reduce meat consumption [7]. But even in the presence of intentions to reduce meat intake, meat-eating habits still strongly predict future meat consumption [8], which can lead to an intention-behaviour gap [9]. Self-monitoring has been found to be effective in helping individuals reduce their meat consumption [7, 10, 11], perhaps because it highlights current consumption levels and helps individuals to identify where their intake could easily be reduced. A more active approach in helping individuals break their meat-eating habits is to encourage them to experiment with meat reduction actions. Previous studies testing action-planning interventions, often in the form of implementation intentions, have been successful in reducing participants’ meat intake [8, 12].

The self-regulation paradigm combines self-monitoring and action-planning, with goal-setting and a reflection process, allowing individuals to use feedback loops to iteratively approach their goal [13, 14]. It is argued that self-regulation can occur naturally following self-monitoring [10, 11, 13, 14], but guiding individuals through the process has been found to be more effective in previous health behaviour research [15–17]. The present study evaluated the effectiveness of an online self-regulation intervention, which aims to support individuals in self-monitoring their meat consumption, trialling different meat reduction actions, and reflecting on the usefulness and effectiveness of these actions based on self-monitoring and educative feedback.

## Methods

### Study design and setting

We conducted an individually randomised, two-arm parallel trial to test the effectiveness of the self-regulation intervention OPTIMISE (Online Programme to Tackle Individual Meat Intake through SElf-regulation) to reduce meat intake compared to a control condition. The study was delivered remotely through a bespoke website developed specifically for the intervention, through which all data collection took place between 15 March and 26 May 2021. The trial was granted ethical approval by the Central University Research Ethics Committee (CUREC) of the University of Oxford (REF: R71398/RE002).

### Participant recruitment

We aimed to recruit 150 participants. The sample size was calculated with the aim to detect a medium-sized effect of *d* = 0.5 between conditions, at 80% power and 5% type 1 error rate, while allowing for a 15% drop-out rate [18]. Participants were recruited through Prolific Academic [19] and completed a screening questionnaire on JISC online surveys [20]. To be eligible, participants had to be aged 18 years or over, resident in the UK, eat meat at least five times per week, indicate they want to reduce their meat intake and be able to engage with the intervention content. Eligible participants who provided consent were invited to register with the OPTIMISE website through Prolific Academic, on a first-come first-served basis. The text from our Prolific Academic study advertisements is provided in Online Resource 1. Depending on adherence to the study procedures, participants were reimbursed with a payment of up to £32 (£0.50 for the screening questionnaire, and £1.50 or £0.75 per session for control group (21 sessions) and intervention group (42 sessions) participants, respectively).

### Randomisation

After indicating their consent, participants were randomly assigned to intervention or control groups using a computer-generated list, with 1:1 randomisation. Participants were blinded to group allocation and informed they would be randomised to one of two groups, each following a different approach for reducing meat intake.

### Study procedures

**Fig. 1:**
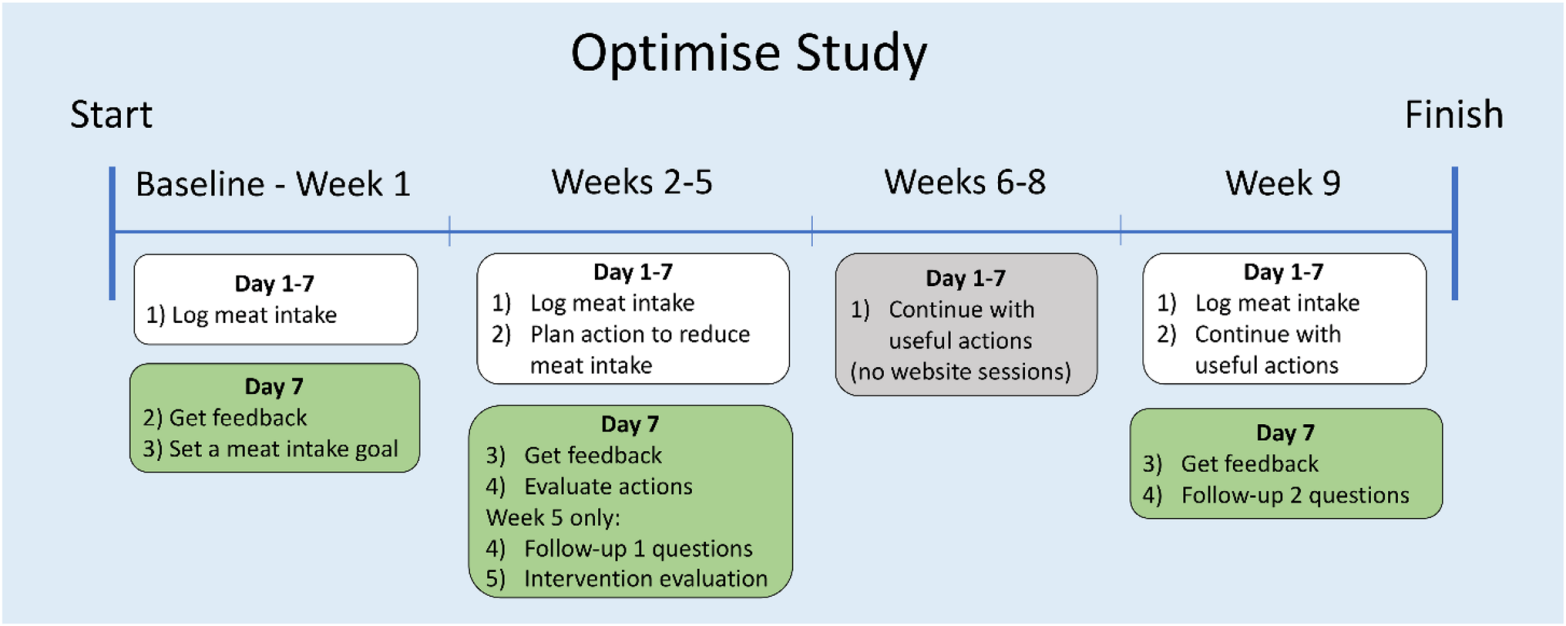
OPTIMISE intervention procedure. Timeline of the 9-week OPTIMISE study, indicating that participants log into the website daily during weeks 1, 2–5 and 9, and have no website sessions during weeks 6–8

The study duration was 9 weeks (a baseline week of self-monitoring meat consumption, a 4-week active intervention phase, and a 4-week maintenance phase; Fig. 1). After registering with the website, all participants were presented with information regarding the health and environmental benefits of eating less meat (Online Resource 2). Participants then completed a baseline questionnaire that asked about their demographic characteristics, meat-free self-efficacy and meat-eating identity. To estimate meat-free self-efficacy, we used a scale adapted from Lacroix & Gifford’s self-efficacy scale [21], where participants were asked to rate the following statements on a scale from 1 (strongly disagree) to 7 (strongly agree): (1) “I lack the cooking skills to prepare meat-free meals”; (2) “I don’t know what to eat instead of meat” and (3) “I don’t have enough willpower to not eat meat”. To capture participants’ meat-eating identity, participants could self-identify as one of six identities (meat eater, omnivore, flexitarian, pescetarian, vegetarian or vegan). Despite our eligibility criteria for participants to eat meat regularly, participants were offered all meat-eating identity options at baseline and both follow-ups, as we know from our public engagement events that meat-eating identities do not always reflect actual consumption.

Meat consumption was measured daily during the baseline week (week 1), first follow-up week (week 5; FU1), and second follow-up week (week 9; FU2) using a meat frequency questionnaire, which has been found to be reliable and acceptable in a UK sample [22]. This questionnaire combines data on food portion sizes from the UK Food Standards Agency with estimates of meat content in composite dishes from the UK National Diet and Nutrition Survey (NDNS). It asks participants to report how many servings of different meat and seafood products they consumed in the previous 24 h.

At each follow-up, participants repeated the meat-free self-efficacy and meat-eating identity questionnaires. At FU2, participants were asked what they thought the other study group had been doing to assess blinding. Participants received automated messages to their Prolific Academic accounts by the OPTIMISE website, prompting them to complete the sessions.

### Intervention group

On the last day of the baseline week, participants received health and environmental feedback on their total meat and red meat consumption. They pre-selected strategies from a list of 26 meat consumption reduction actions (Online Resource 3) and set themselves a meat reduction goal. These strategies were created specifically for this study during a brainstorming session with a group of experts, including nutritionists, psychologists and health behaviour scientists in our department. They were then further refined during Patient and Public Involvement focus group sessions with meat eaters to ensure the actions were appropriate and understandable. The strategies offered a wide range of actions to cater to different stages of meat reduction. Participants were asked to pick actions they found challenging, thus creating their personal set of meat reduction tools. Throughout weeks 2–5, they planned a meat reduction action daily specifying when and how they would perform the action, as well as considering how to overcome barriers. Each subsequent morning they were asked whether they had managed to perform the action they had chosen on the previous day and if not they were asked to reflect on what they could do differently next time. Participants received weekly feedback on how their meat consumption compared to week 1 in terms of quantity and environmental and health impacts (Online Resource 4). Based on this information, they were asked to reflect on the usefulness of the actions they had attempted that week. Following week 5, participants entered a four-week maintenance phase (weeks 6–9), where they were asked to continue performing the actions they found useful during the intervention phase. Participants received access to downloadable materials, such as a detailed overview of the meat reduction actions and an action diary, to provide the opportunity to engage with the intervention offline. At FU1, participants completed an intervention evaluation questionnaire.

### Control group

After the baseline week, participants were asked to try to reduce their meat consumption during the following eight weeks with no further guidance. At FU2, participants completed a questionnaire and were asked what strategies they had tried to reduce their meat consumption.

### Outcomes

#### Primary outcome

The primary outcome was the difference between groups in change in total daily meat consumption from baseline to FU1, based on 7-day self-reported meat intake in weeks 1 and 5.

#### Secondary outcomes

The difference between groups in change in meat consumption from baseline to FU2, and from FU1 to FU2 was analysed as secondary outcomes. In addition, from baseline to both follow-ups, we compared the difference between groups in: (i) change in consumption of meat sub-types (red meat, processed meat, red and processed meat combined); (ii) change in meat-free self-efficacy; and (iii) change in meat-eating identity. We also assessed differences in the effect of the intervention on our primary outcome by meat-free self-efficacy level.

We explored the acceptability and feasibility of the intervention for reducing meat consumption through intervention evaluation questionnaire responses collected at FU1 and adherence throughout the study, respectively. Barriers to adherence to self-selected meat reduction actions were explored through the free-text responses to the daily action completion question, which was asked when respondents indicated they had not been able to perform their action during weeks 2–5 (active intervention period). Strategies reported by the control group participants in the strategy exploration questionnaire administered at FU2 were compared to actions taken by the intervention group from the suggested action list.

#### Statistical analysis

Quantitative analyses were conducted in Stata/IC Version 14.1. Qualitative data were coded and managed using NVivo 12 software. We published a statistical analysis plan on the Open Science Framework (28/04/2021) preceding the analyses [23].

For each participant and time point (baseline, FU1, and FU2), we calculated mean total daily intakes of meat, red meat, processed meat, and red and processed meat combined, and mean meat-free self-efficacy scores. Participants were grouped into tertiles of baseline self-efficacy (low-/medium-/high- self-efficacy). Meat-eating identities were grouped into three higher-level categories: (i) non-meat-eating identity; (ii) reduced meat-eating identity; (iii) meat-eating identity. We created a dummy variable for ‘positive meat-identity change’ for both follow-ups. This was coded as 1 if a positive meat-identity change had occurred or 0 if a positive meat-identity change had not occurred.

#### Primary analysis

The member of the research team analysing the primary outcome was blinded to group allocation. A linear regression model was used to determine whether the change in mean daily meat intake from baseline to FU1 differed significantly between the intervention and control groups.

#### Secondary analyses

Linear regression models were also used to explore changes in: (i) meat intake from baseline to FU2; (ii) meat intake from FU1 to FU2; (iii) intake of meat sub-types (red meat, processed meat, and red and processed meat) from baseline to both follow-ups; and (iv) participants’ meat-free self-efficacy scores from baseline to both follow-ups. We also explored differences in the effect of the intervention on our primary outcome by baseline self-efficacy by introducing self-efficacy tertiles as a predictor in the model. We employed a logistic regression model to determine whether the intervention increased participants’ odds of making a positive meat-eating identity change at both follow-ups. Written feedback collected from participants as part of the evaluation questionnaire, strategy exploration questionnaire and daily action completion questions were analysed qualitatively using inductive thematic analysis [24], with all responses coded and then grouped into broader categories of shared meaning.

#### Exploratory analyses

We carried out a sensitivity analysis excluding days on which participants’ total meat intake exceeded 1.5 kg to assess the effect of outliers. All participants in our final sample completed > 4 meat frequency questionnaires during the baseline week and FU1 so we did not carry out the second sensitivity analysis stated in our pre-published statistical analysis plan.

## Results

### Participants

**Fig. 2:**
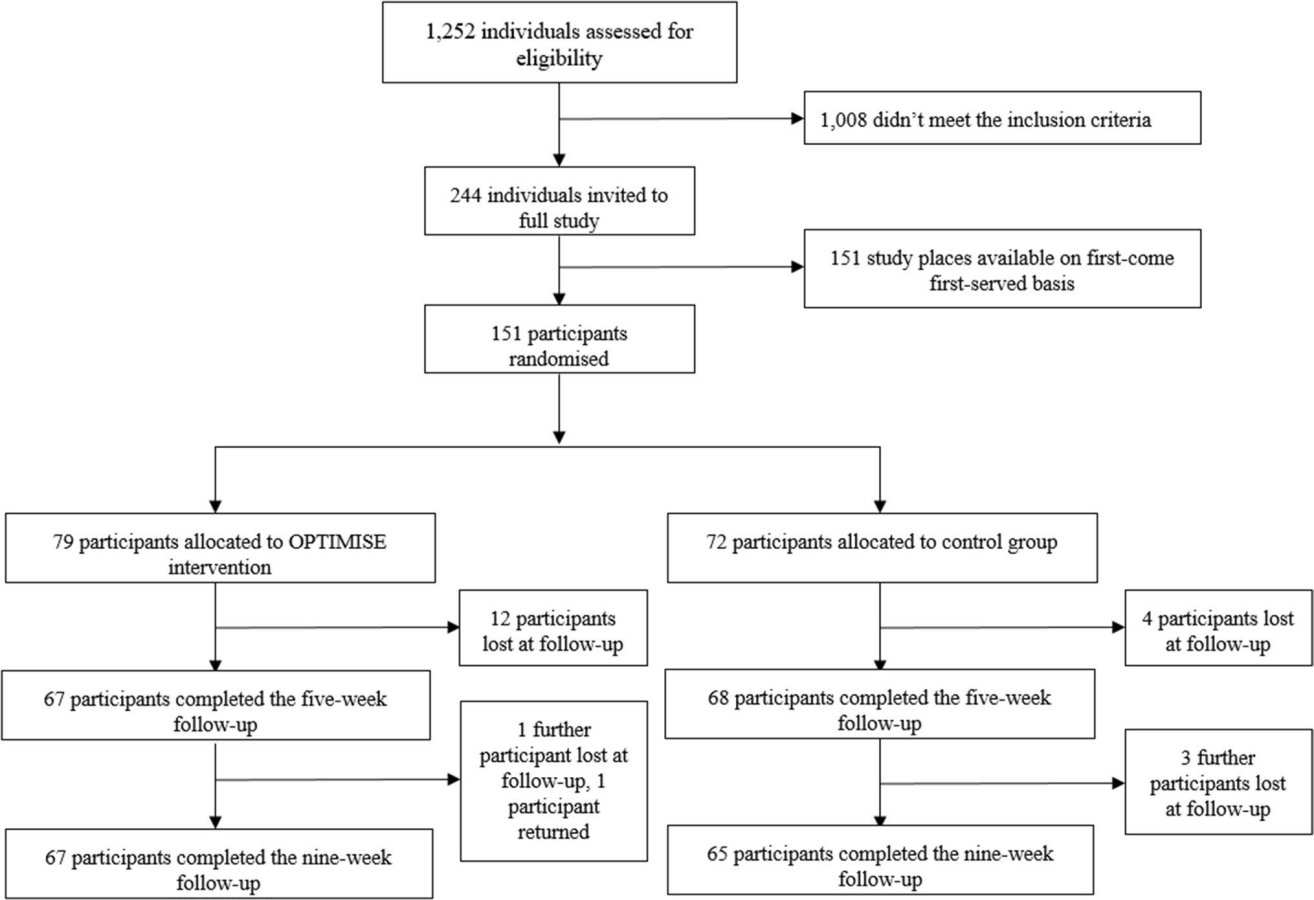
Study flowchart of the OPTIMISE trial

We approached 1252 participants, 244 were eligible and invited to take part in the full study. We randomised 151 participants on a first-come, first-served basis (Fig. 2). Of these, 79 were allocated to the intervention group and 72 to the control group. Participants were aged 18–67 years (mean 37.1 ± 11.9 years), 62% were female, 76% were white British, 64% had a university degree and 62% were employed (Table 1). Mean baseline total meat consumption was 226 g/day (221 g/day in the intervention group and 231 g/day in the control group; Table 2).

**Table 1:** Baseline demographics (*n* = 151)

		Control *N* = 72	Intervention *N* = 79	Total *N* = 151
Age, mean (SD)		35.7 (11.4) (min–max: 19–67)	38.4 (12.3) (min–max: 18–67)	37.1 (11.9) (min–max: 18–67)
Gender, *n* (%)	Female	42 (58.3)	51 (64.6)	93 (61.6)
	Male	30 (42.7)	27 (34.2)	57 (37.8)
	Other	0 (0.0)	1 (1.3)	1 (0.7)
Ethnicity, *n* (%)	White-British	53 (73.6)	62 (78.5)	115 (76.2)
	White-Other	10 (13.9)	9 (11.4)	19 (12.6)
	Asian or Asian-British	5 (6.9)	4 (5.1)	9 (6.0)
	Black or Black-British	2 (2.8)	0 (0.0)	2 (1.3)
	Mixed/Other	2 (2.8)	4 (5.1)	6 (4.0)
Region of the UK, *n* (%)	Greater London	9 (12.5)	9 (11.4)	18 (12.0)
	East of England	9 (12.5)	6 (7.6)	15 (10.0)
	South East	13 (18.1)	10 (12.7)	23 (15.2)
	South West	2 (2.8)	7 (8.9)	9 (6.0)
	East Midlands	10 (13.9)	7 (8.9)	17 (11.3)
	West Midlands	5 (6.9)	12 (15.2)	17 (11.3)
	Yorkshire and the Humber	6 (8.3)	6 (7.6)	12 (8.0)
	North West	7 (9.7)	9 (11.4)	16 (10.6)
	North East	3 (4.2)	1 (1.3)	4 (2.7)
	Scotland	5 (6.9)	7 (8.9)	12 (8.0)
	Wales	3 (4.2)	5 (6.3)	8 (5.3)
	Northern Ireland	0 (0.0)	0 (0.0)	0 (0.0)
Highest educational qualification, *n* (%)	University degree, NVQ level 4–5 or equivalent, and above	50 (69.4)	46 (58.2)	96 (63.6)
	Other post high school qualifications	2 (2.8)	4 (5.1)	6 (4.0)
	A’ levels, NVQ level 2–3 or equivalent	11 (15.3)	23 (29.1)	34 (22.5)
	Apprenticeship	0 (0.0)	2 (2.5)	2 (1.3)
	GCSE, NVQ level 1, or equivalent	9 (12.5)	2 (2.5)	11 (7.2)
	Other vocational, work-related qualifications	0 (0.0)	2 (2.5)	2 (1.3)
	No formal qualifications	0 (0.0)	0 (0.0)	0 (0.0)
Employment^a^, *n* (%)	Employed	48 (66.7)	45 (57.0)	93 (61.6)
	Self-employed	5 (6.9)	7 (8.9)	12 (7.9)
	Unemployed	4 (5.6)	4 (5.1)	8 (5.3)
	Looking after home or family	5 (6.9)	13 (16.5)	18 (11.9)
	Student	12 (16.7)	10 (12.7)	22 (14.6)
	Retired	4 (5.6)	5 (6.3)	9 (6.0)
	Long-term sick or disabled	1 (1.4)	3 (3.8)	4 (2.6)

^a^Participants could select multiple answers

**Table 2:** Meat consumption and attitudinal measures at baseline and both follow-ups

	Baseline, *n* = 151		Follow-up 1 (5 weeks), *n* = 135					Follow-up 2 (9 weeks), *n* = 132				
	Control (*n* = 72)	Intervention (*n* = 79)	Control (*n* = 68)	Intervention (*n* = 67)	Difference (adjusted for baseline)			Control (*n* = 65)	Intervention (*n* = 67)	Difference (adjusted for baseline)		
Meat consumption (g/day)	Mean (SD)		Mean (SD)		Mean	95% CI	*P* value	Mean (SD)		Mean	95% CI	*P* value
Total meat	231 (207)	221 (204)	138 (97)	96 (78)	− 40	− 11.6, − 67.5	0.006	122 (97)	107 (107)	− 12	19.1, − 43.4	0.443
Red meat	106 (122)	101 (104)	66 (77)	39 (38)	− 27	− 6.3, − 46.8	0.011	55 (59)	42 (51)	− 13	5.7, − 31.6	0.172
Processed meat	60 (52)	50 (45)	36 (36)	25 (27)	− 7	2.4, − 16.8	0.140	34 (37)	29 (37)	− 2	9.6, − 13.7	0.730
Red and processed meat	166 (151)	151 (130)	102 (102)	64 (59)	− 35	− 7.7, − 61.7	0.012	89 (84)	71 (81)	− 16	11.6, − 42.7	0.259
Total meat—outlier sensitivity	202 (127)	200 (167)	131 (80)	96 (78)	− 33	− 8.5, − 56.8	0.008					

Attitudinal measures	Mean (SD)		Mean (SD		Mean	95% CI	*P* value	Mean (SD)		Mean	95% CI	*P* value
Meat-free self-efficacy score^a^	4.0 (1.3)	3.7 (1.2)	3.6 (1.3)	3.6 (1.4)	0.2	0.57, − 0.17	0.280	3.1 (1.2)	3.1 (1.4)	0.19	0.62, − 0.25	0.398
Meat-eating identity	*N* (%)		*N* (%)					*N* (%)				
Meat-eater	63 (87.5%)	70 (88.6%)	53 (77.9%)	51 (76.1%)	Odds ratio for adopting a positive meat-identity change: or 0.66; 95% CI 0.22, 2.01; *P* = 0.459			43 (66.2%)	42 (62.7%)	Odds ratio for adopting a positive meat-identity change: or 1.00; 95% CI 0.40, 2.52; *P* = 0.995		
Meat-reducer	9 (12.5%)	9 (11.4%)	14 (20.6%)	11 (16.4%)				15 (23.1%)	16 (23.9%)			
Non-meat-eater	0 (0.0%)	0 (0.0%)	0 (0.0%)	0 (0.0%)				0 (0.0%)	1 (1.5%)			
Missing	0 (0.0%)	0 (0.0%)	1 (1.5%)	5 (7.5%)				7 (10.8%)	8 (11.9%)			

^a^Meat-free self-efficacy score was on a Likert scale from 1 to 7

*SD* Standard deviation, *CI* Confidence interval, *OR* Odds ratio

Sixteen participants did not complete FU1 (twelve in the intervention group and four in the control group) and a further four did not complete FU2 (one intervention and three control), though one participant who did not complete FU1 in the intervention group, returned (Fig. 2).

### Changes in meat intake

Across the whole sample, mean meat intake was 118 g/day at five weeks and 114 g/day at nine weeks. Total meat intake decreased by 125 g/day in the intervention group at FU1, 40 g/day more than in the control group (95% CI − 11.6, − 67.5, *P* = 0.006; Table 2). At FU2, mean meat intake in the intervention group was 114 g/day below baseline, but there were no significant differences in total meat reduction between groups (− 12 g/day intervention vs control, 95% CI 19.1, − 43.4, *P* = 0.443; Table 2). There was no significant difference in change in total meat intake from FU1 to FU2 between groups (16 g/day intervention vs control, 95% CI 44.2, − 12.9, *P* = 0.281). The intervention led to a 27 g/day reduction in red meat (95% CI − 6.3, − 46.8, *P* = 0.011) and a 35 g/day reduction in red and processed meat combined (95% CI − 7.7, − 61.7, *P* = 0.012) at FU1, in comparison to the control group. No significant reductions between groups were observed at FU2 for individual meat sub-types (Table 2).

Total meat intake reported by 12 participants on 16 individual days (14 baseline, 2 FU1) exceeded 1.5 kg/day. There were no exclusions at FU2. In our pre-planned sensitivity analysis, exclusions of these days did not materially change the findings (Table 2).

### Change in attitudinal measures

There was no evidence that the intervention changed meat-free self-efficacy scores, and the effectiveness of the intervention did not differ by baseline self-﻿efficacy. A small number of participants made a positive meat-eating identity change over time, but this did not differ between groups (Table 2).

### Actions taken by intervention and control group participants

All 26 meat reduction actions offered as part of the intervention were chosen at least once (Online Resource 5). The following six were chosen more than 100 times: make at least one of your main meals vegetarian (*n* = 257); double the veg, halve the meat (*n* = 187); eat no red meat (*n* = 183); set yourself a maximum number of animal products to consume today (*n* = 149); eat no processed meat (*n* = 123); and try a new vegetarian recipe (*n* = 103). The action “go plant-based for the whole day” was the least popular action and chosen only twice.

Fifty-nine (91%) control group participants completed the questionnaire about strategies they had used to reduce their meat consumption. In total, they reported 16 different strategies, 10 of which were similar to those chosen by the intervention group participants. The top five strategies were also offered as part of the intervention: (i) try a meat-alternative (*n* = 26); (ii) try new vegetarian recipes (*n* = 23); (iii) have meat-free meals (*n* = 20); iv) reduce the proportion of meat in composite dishes (*n *= 19); and (v) have a meat-free day (*n* = 7). Other strategies chosen more than once that were not offered as part of the intervention were: (i) planning and preparing meals in advance; and (ii) subscribing to recipe delivery boxes.

### Self-reported barriers

**Fig. 3:**
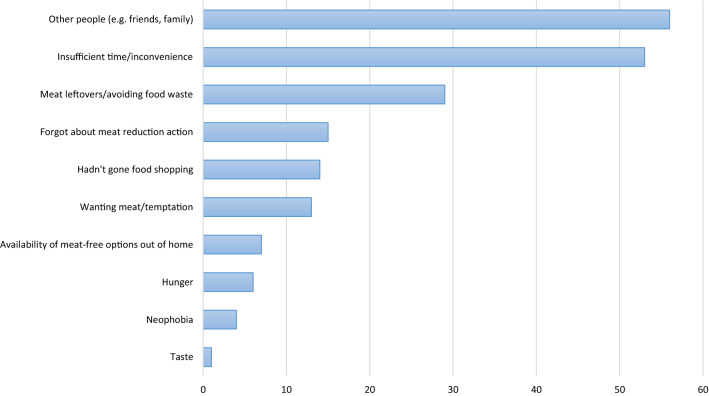
Participants self-reported barriers for not adhering to their planned daily meat reduction actions. Results from open-text responses to the daily (weeks 2–5) action completion question which were collected when participants indicated they had not been able to perform their action: “please tell us a little bit more about why you were unable to stick to the action you had planned.” Horizontal bar graph depicting the frequency of self-reported barriers. Ten barriers are displayed: other people (e.g. family and friends, *n* = 56), insufficient time/inconvenience (*n* = 53), wanting to avoid food waste/eating meat leftovers (*n* = 29), forgetting about the action (*n* = 15), had not gone food shopping (*n* = 14), wanting meat/temptation (*n* = 13), availability of meat-free options out of home (*n* = 7), hunger (*n* = 6), neophobia (*n* = 4) and taste (*n* = 1)

The most common reasons provided by intervention participants for not being able to perform their planned daily meat reduction actions were: other people (e.g. family and friends, *n* = 56), insufficient time/inconvenience (*n* = 53) and wanting to avoid food waste/eating meat leftovers (*n* = 29). See Fig. 3.

“I put meat in my vegetarian meal, my husband’s face dropped when I told him the meal and so I told him I would put ham in it.”

“I haven’t yet thought about what vegetarian meal to cook for the week. I was dealing with work, homework and with builders in the building and so taking time to think of an extra unusual meal is difficult to deal with.”

“Unfortunately there were meat leftovers from dinner a couple of nights ago in the fridge, and my partner pointed out if we didn’t use it last night it would have to be thrown away, and I really hate wasting food.”

### Acceptability and feasibility of the intervention

Adherence was high with 80% of intervention participants and 89% of control participants completing at least 80% of their sessions (66% and 68% of intervention and control participants completed all respective sessions). Sixty-three intervention participants (80%) completed the intervention evaluation questionnaire and rated the usefulness of the intervention components and the additional resources on a scale of 1 (not useful) to 10 (very useful). Feedback was largely very positive with mean scores ranging from 7.4 to 8.5 (Table 3). Participants found tracking their meat consumption daily to be the most useful part of the intervention (mean score 8.5 ± 1.7) and the ability to review their journey was found to be the most useful additional resource available (mean score 8.5 ± 1.4). Only two participants did not provide feedback in response to a free text question. The large majority of responses were positive.

“This is a great study and motivated me to decrease my meat intake and include more healthy vegetarian options in my meals”

“Very detailed, informative and fascinating information.”

“I have found this study to be a real eye opener with my meat consumption and the effect on the planet”

“Thank you because I never thought I'd be able to live without bacon and I don't even miss it!”

**Table 3:** Intervention evaluation questionnaire results

How useful did you find On a scale from 1 (not useful) to 10 (very useful)	Mean (SD)	N
Intervention components		
“Tracking your meat consumption on a daily basis?”	8.5 (1.7)	63
“The feedback on the environmental and health impact of your meat consumption?”	8.0 (2.4)	63
“Planning an action on a daily basis to reduce your meat consumption?”	7.8 (2.1)	63
Additional resources		
“The weekly action evaluation?”	8.2 (1.7)	63
“The downloadable Action Diary?”	8.3 (1.5)	6
“The downloadable Action Overview?”	7.4 (1.3)	8
“The links to other resources?”	7.5 (1.6)	13
“The ability to review your journey?”	8.5 (1.4)	38

Results from the intervention evaluation questionnaire administered at FU1 (week 5) to intervention participants. The additional resources were optional and were only evaluated by those who reported using them throughout the study

Five participants reported that some of the meat reduction actions were difficult to do daily as they required planning, and two participants found filling out the meat frequency questionnaire difficult.

When asked what they thought the other group were doing, the majority of participants in both control and intervention groups said they were not sure, and so we concluded that blinding was successful.

## Discussion

The OPTIMISE self-regulation intervention was designed to support people in reducing their meat consumption. In a group of frequent meat consumers, there was a large reduction in meat intake at both follow-ups, including a reduction in red and processed meat, which was significantly greater than that of the control group after five weeks. The difference between groups was not sustained after the 4-week maintenance phase, with both groups reducing their meat intake to a similar level. There was no evidence that the intervention increased participants’ meat-free self-efficacy nor participants’ odds of making a positive meat-eating identity change. The evaluation and adherence throughout revealed the intervention was acceptable and feasible with the majority of participants finding the intervention informative and enjoyable.

We are unable to definitively identify which components contributed most to the initial significant reduction in meat intake in the intervention group (− 125 g/day at FU1) due to the multi-component nature of the OPTIMISE programme. We speculate that a large proportion of the effect came from self-monitoring due to the reductions also observed in the control group, who were required to self-monitor their meat consumption daily during weeks 1, 5 and 9. The additional intervention effect that we found at FU1 (− 40 g/day vs control) can be assigned to a combination of the other components of OPTIMISE. In contrast to the control condition, self-monitoring in the intervention condition continued throughout weeks 2–5, allowing individuals to follow changes closely over time, likely enhancing the effectiveness of this strategy. The intervention group was also asked to set themselves a meat reduction goal, and the combination of this with self-monitoring has been found to be more effective at promoting dietary behaviour change than either component alone [25]. In previous research, a multi-component intervention utilising goal-setting, self-monitoring and an informational/educational component was effective at reducing meat consumption in young men [26]. In the current study, we also included educational components, which were tailored to the individuals’ self-monitored consumption. Perhaps most importantly, the OPTIMISE intervention asked individuals to plan daily actions to break their meat-eating habits. Action-planning after goal-setting has also been found to be effective in other studies that tested meat reduction interventions [8, 12]. Taken together, our results suggest that formally guiding individuals through the whole self-regulation process (goal-setting, self-monitoring, and action-planning with regular reflection) is more effective in the short term than prompting individuals to self-monitor alone and relying on natural self-regulation, in line with other health behaviour research [15–17].

Despite the significant effect of the intervention at FU1, there were no additional benefits of the intervention on meat reduction compared to control at FU2. In fact, the data indicate that the lack of guidance in the maintenance phase of the intervention led to a reduction in self-regulation compared to FU1, as there was a lower meat reduction effect at FU2 (− 125 g/day at FU1 to − 114 g/day at FU2). In the control group, self-monitoring during weeks 1, 5, and 9 seemed to continue to prompt a gradual increase in the level of natural self-regulation (− 93 g/day at FU1 to − 109 g/day at FU2) [14]. It is possible that the active intervention components in the intervention group did not have a long-lasting effect, and meat reductions at FU2, therefore, relied on natural self-regulation, similar to the control group.

As also speculated above, the size of the reductions at FU2 indicates that self-monitoring has a large effect on its own—the meat consumption reductions equated to 52% and 47% in intervention and control groups, respectively. For context, a trend analysis of the UK NDNS found that meat intake has decreased by only 17% over a recent ten year period (2008/09–2018/19) [4]. In 2010, the UK Scientific Advisory Committee on Nutrition set the target that adults with high intakes of red and processed meat (> 90 g/day) should limit their intakes to a maximum of 70 g/day [27]. Both groups exceeded this recommendation at baseline (151 g/day and 166 g/day in intervention and control groups, respectively), but intervention group participants met this recommendation at FU1 (64 g/day vs 102 g/day in the control group). At FU2, mean consumption of red and processed meat was 71 g/day in the intervention and 89 g/day in the control group. The significant size of the reduction could—to some extent—reflect the high intrinsic motivation of participants to reduce their meat consumption, given our study eligibility criteria that participants had to indicate they wanted to reduce their meat intake. It is also possible that what we have ascribed to self-regulatory changes here, may also be measurement reactivity, a bias regularly observed in trials [28], or intentional underreporting, for example, due to social desirability. However, the theory that natural self-regulation may have led to these long-term reductions in both groups is also in line with other research showing that asking individuals to self-monitor meat consumption significantly changes it [10, 11]. Indeed, despite not having had access to intervention materials, control group participants reported spontaneously adopting similar meat reduction strategies to those chosen as part of the OPTIMISE intervention.

The lack of long-term effects of the active components of the intervention could be ascribed to the fact that the OPTIMISE intervention only targeted individuals’ reflective decision-making processes, although most of our human decision-making, including meat-eating, is through the unconscious and automatic response system, based on conditioning and heuristics [8]. Behaviour change on these automatic responses can be accomplished through repetition, but the OPTIMISE intervention was likely too short to achieve this and individuals, therefore, tended to slip back into their old routines. The concept that behaviour change should be considered a dual process has been reported previously [2], and it could be hypothesised that individual-level, conscious, meat reduction interventions would be more effective in the presence of a supportive environment including interventions operating through sub-conscious mechanisms to achieve long-term change.

The lack of difference between groups at FU2 is consistent with the lack of effect of the intervention on meat-free self-efficacy. Greater perceived self-efficacy has been associated with the adoption of desirable healthy lifestyle behaviours [29, 30] including eating less meat [31]. It may be that the duration of the active intervention (4 weeks) was insufficient to impact participants’ self-efficacy. Another explanation may be that not all of our meat reduction actions were sufficiently geared towards improving the types of self-efficacy that we measured. That is, actions, such as “avoid the meat and fish aisle when shopping” and “eat no processed meat”, might not have helped participants improve their self-efficacy on cooking meat-free meals, knowing what to eat, or having the willpower to not eat meat. Our intervention also did not increase participants’ odds of making a positive meat-eating identity change. Several participants mentioned in their free-text evaluation responses that self-monitoring their meat consumption was an eye-opener. Accordingly, it is possible that during the study, participants realised they consumed more meat than they had previously thought, and reassessed their meat-eating identity, obscuring any effect of the intervention.

A strength of this study is the use of a website to manage all data collection, avoiding any direct contact with the researcher and reducing the risk of researcher bias. The use of our recently developed meat frequency questionnaire allowed us to reliably measure changes in participants’ meat intake at a granular level [22] and measuring intake over seven days at each assessment point allowed us to better estimate habitual meat intake, which can vary considerably day to day. Allocation concealment in the randomisation process and blinding of the researcher who analysed our primary outcome helped to further reduce bias, and we concluded from the results of our debriefing questionnaire that participants were unaware of their group allocation. Underreporting is an inherent limitation of self-reported dietary assessment methods [32], though we hope our detailed meat frequency questionnaire and focus on changes in intake as opposed to absolute intakes helps alleviate this limitation. Similarly, while measuring meat intake daily for seven days was a strength, our observed meat reduction in the control group suggests it acted as an active comparator intervention and thus we didn’t have a true ‘no-intervention’ control. It is important to note also that this study was only powered at 80% to find a medium-sized effect, and this may not have been sufficient to detect an effect at FU2. Another limitation is that 1008 participants did not meet our eligibility criteria and unfortunately, JISC Online Surveys did not allow us to view the reasons for exclusion. Moreover, we did not capture the sources of participants’ motivations for wanting to reduce their meat consumption. As participants were recruited through Prolific Academic, it is possible they were motivated by the financial reward, but any bias resulting from this would have been equal across groups. Future research may wish to test a similar self-regulation intervention among a more general population sample and try to measure their meat reduction motivations. Our participants were high meat-eaters (consuming meat at least five times per week) and the effect of the intervention among low- /moderate- meat-eaters is unknown. For context, we observed a baseline meat consumption of 221 g/day and 231 g/day in intervention and control groups, respectively, while the average meat intake, including fish, per consumer in the UK was reported to be 127 g/day in 2019 [4]. Future research testing the OPTIMISE intervention with low- /medium- meat-eaters would be useful to determine whether the intervention can be generalised to a broader population sample.

## Conclusion

Our results indicate that this online multi-component self-regulation intervention was effective for reducing meat intake in the short term among high meat-eating UK adults. The subgroup analysis on red and processed meat, which have the biggest environmental and health impacts, also showed significant reductions. Participants found the intervention to be useful and enjoyable and coupled with high adherence rates we conclude the intervention is feasible and acceptable. Specific effects of the intervention in the longer term were uncertain though meat intake in both groups decreased considerably suggesting the combination of educative components and self-monitoring meat consumption may have constituted an active comparator intervention.

## Supplementary Information

Below is the link to the electronic supplementary material.

Supplementary file1 (DOCX 17 KB)

Supplementary file2 (DOCX 428 KB)

Supplementary file3 (DOCX 21 KB)

Supplementary file4 (DOCX 301 KB)

Supplementary file5 (DOCX 22 KB)

## Data Availability

The datasets used and/or analysed during the current study are available from the corresponding author on reasonable request.
