# Supplementary material for: Effectiveness of an Online Programme to Tackle Individual’s Meat Intake through SElf-regulation (OPTIMISE): A randomised controlled trial
Source: Eur J Nutr. 2022 Mar 4;61(5):2615–26. doi: 10.1007/s00394-022-02828-9 (PMC9279210; doi:10.1007/s00394-022-02828-9)
Supplement: Supplementary file 1 — Supplementary file1 (DOCX 17 KB) [file 394_2022_2828_MOESM1_ESM.docx]

**SI 1.** Study advertisement text on Prolific Academic

**Advertisement for screening questionnaire**

*Study title*

Screening for The Optimise Study – Online Programme to Tackle Individual’s Meat Intake through SElf-regulation

*Study Description*

We’re a group of researchers at the University of Oxford who are interested in helping individuals make healthier and more environmentally-friendly food choices. In this study (called Optimise) we want to find out how we can help people who want to reduce their meat consumption to do so.

The study will last 9 weeks and can be completed online on our Optimise website. We estimate that as a participant you may spend a total of 6 hours on the study tasks over the course of the 9 weeks. You will be reimbursed for this time through your Prolific account. If you decide to take part, you will be randomised to one of two groups at the first session. Each group will follow a different approach to reducing meat intake for the following 9 weeks. Depending on which approach you follow, you may spend up to 10 minutes every day on the programme tasks. Rest assured, you will not be asked for any information that could identify you.

Does this sound like something you’d like to try? Find out whether you are eligible to take part in the Optimise study by completing this screening survey. It will take you 5 minutes to complete and you will be reimbursed for your time.

**Advertisement to register with the study website - seen by eligible participants only and 151 spaces were available on a first-come first-served basis**

*Study title*

First Session Optimise Study – Online Programme to Tackle Individual’s Meat Intake through SElf-regulation

*Study Description*

We’re a group of researchers at the University of Oxford who are interested in helping individuals make healthier and more environmentally-friendly food choices. A few days ago you completed our screening questionnaire to find out whether you could take part in our Optimise study. In this study we want to find out how we can help people who want to reduce their meat consumption to do so. As you are eligible for our study, we would like to invite you to take part in our programme and reduce your meat intake with us.

The study lasts 9 weeks and can be completed online on our Optimise website. We estimate that you may spend a total of 6 hours on the study tasks over the course of the 9 weeks. You will be reimbursed for this time through your Prolific account. For today, we would like to invite you to sign up to the study and take part in our 30-minute long first session. If you decide to take part, you will be randomised to one of two groups today. Each group will follow a different approach to reducing meat intake for the next 9 weeks. Depending on which approach you follow, you may spend up to 10 minutes every day on the programme tasks. Rest assured, you will not be asked for any information that could identify you. Would you like to take part? If so, please click on the link below. This is a personalised link, so please do not share it with anybody else.
