# Supplementary material for: Effectiveness of an Online Programme to Tackle Individual’s Meat Intake through SElf-regulation (OPTIMISE): A randomised controlled trial
Source: Eur J Nutr. 2022 Mar 4;61(5):2615–26. doi: 10.1007/s00394-022-02828-9 (PMC9279210; doi:10.1007/s00394-022-02828-9)

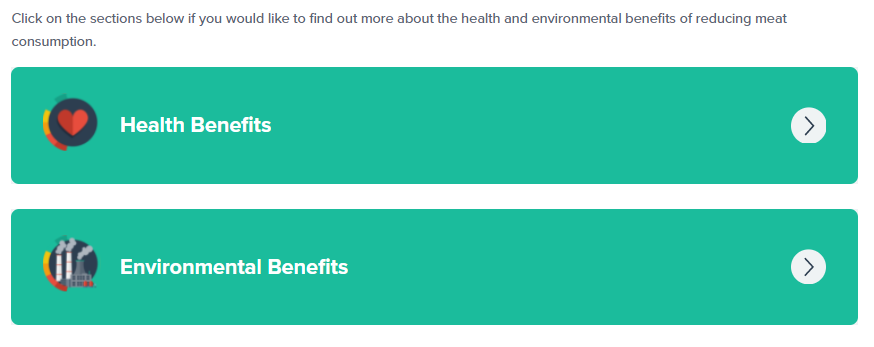

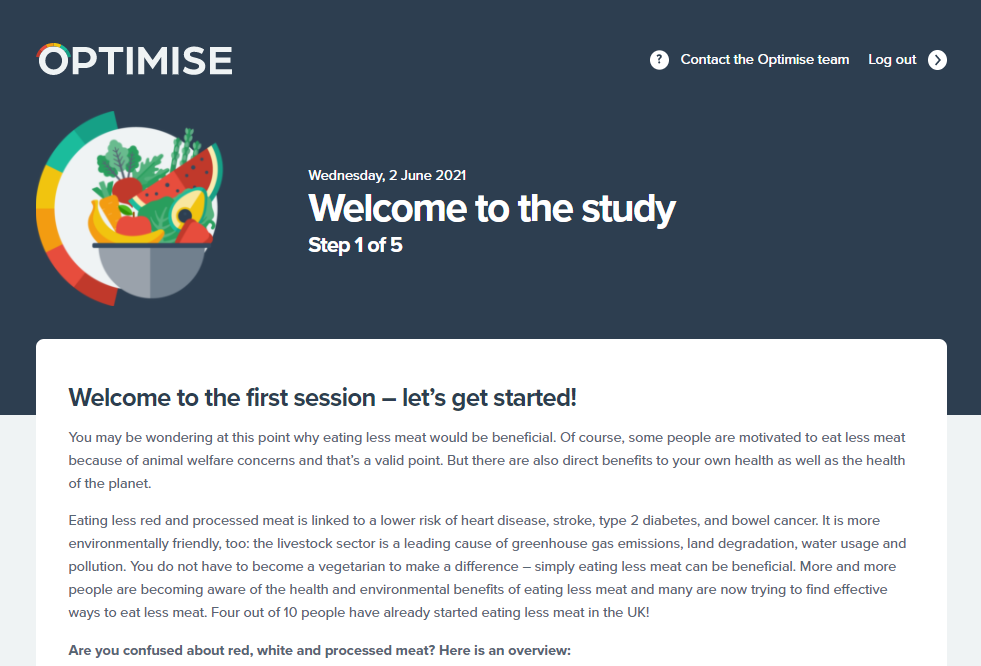
**SI 2.** Health and environmental information presented to participants when they first registered with the OPTIMISE study website


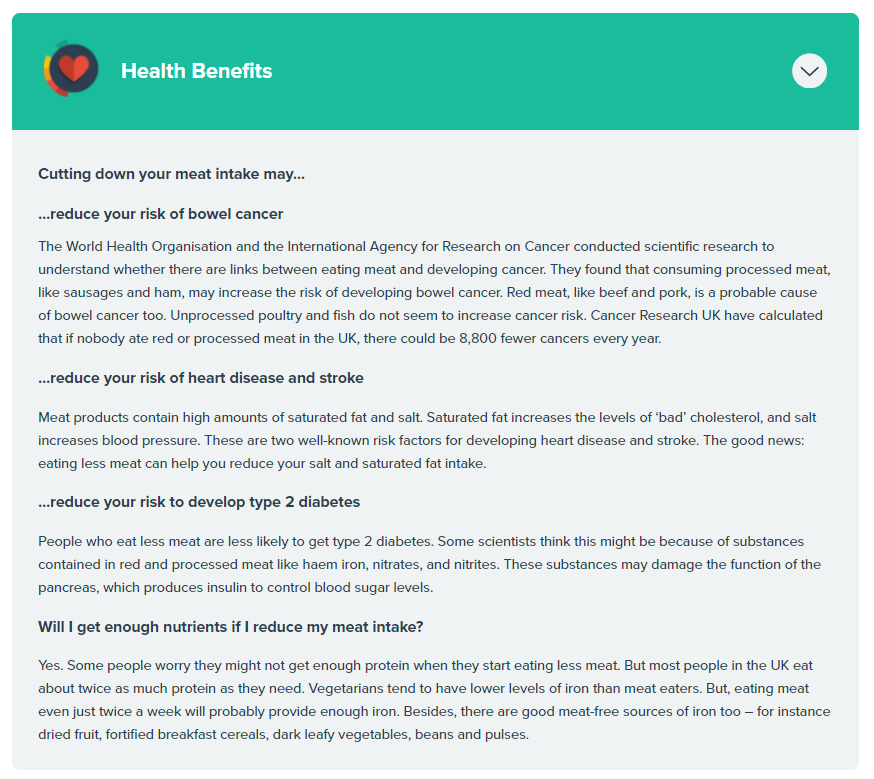


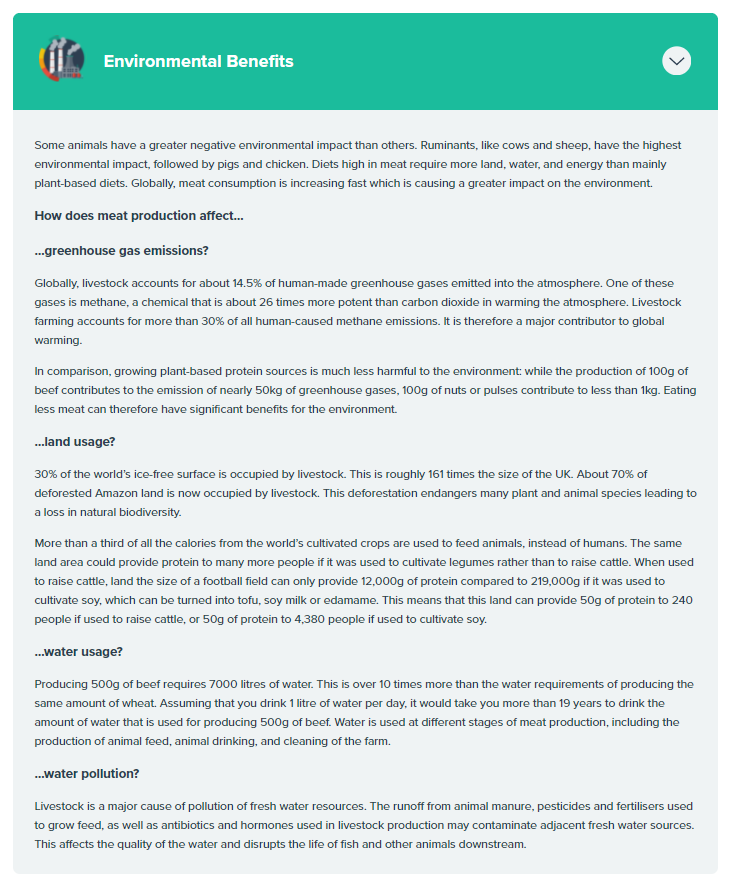

Supplement: Supplementary file 2 — Supplementary file2 (DOCX 428 KB) [file 394_2022_2828_MOESM2_ESM.docx]
