# Supplementary material for: Effectiveness of an Online Programme to Tackle Individual’s Meat Intake through SElf-regulation (OPTIMISE): A randomised controlled trial
Source: Eur J Nutr. 2022 Mar 4;61(5):2615–26. doi: 10.1007/s00394-022-02828-9 (PMC9279210; doi:10.1007/s00394-022-02828-9)
Supplement: Supplementary file 3 — Supplementary file3 (DOCX 21 KB) [file 394_2022_2828_MOESM3_ESM.docx]

**SI 3.** Daily meat reduction actions offered as part of the OPTIMISE intervention

| **Category** | **Actions** | **What to do** |
| --- | --- | --- |
| **Preparing for change** | 1. Set yourself a maximum number of animal products to consume today and stick to it | Start the day by deciding on the maximum number of animal-derived products you want to allow yourself to eat today. For example, you might decide that you want to restrict yourself to 3 animal-derived products. This would allow you to have milk in your tea in the morning, some cheese during lunch and some bacon in your dinner. The rest of the day, go plant-based. Make sure to set yourself a challenge. If you succeed at sticking to your goal, set yourself a more ambitious goal next time. |
|  | 1. Don’t buy any food containing meat | Make sure not to buy any food that contains meat. Remember that meat can be a hidden ingredient, so make sure to also refrain from foods such as ham sandwiches, or pizza with meat toppings. |
|  | 1. Buy lots of (fresh or frozen) vegetables and fruit to make sure you have enough to bulk up meals | Go to the supermarket and buy fresh or frozen vegetables, cans of pulses, fruits and nuts so that you don’t struggle with cooking more meat-free dishes in the next couple of days. |
|  | 1. Avoid the meat and fish aisle when shopping | When you go shopping, make sure not to walk down the meat and fish aisle – this will reduce your exposure to temptations. |
|  | 1. Plan a vegetarian day for this week | Make a meal plan that doesn’t include any meat products for a day later this week. Make sure to plan food from breakfast to dinner, including snacks. You might even want to look up new vegetarian recipes to make things more interesting. |
|  | 1. Read ingredient lists to identify what is vegetarian/plant-based | Read the ingredient lists of things you want to buy, prepare, or eat. A lot of non-vegetarian and non-plant-based ingredients show up in unexpected places, such as gelatine in sweets or dairy products in baked goods. By reading ingredient lists, you will learn a lot about where animal-derived products are hidden, and where, sometimes surprisingly, there are none. |
| **Try swapping out meat for veg** | 1. Double the veg, halve the meat | For all your meals today, have only half the amount of meat you usually eat, and instead fill the plate with vegetables, pulses or legumes. |
|  | 1. Take a classic meat dish and turn it vegetarian | Choose a meat dish and see how you can make it vegetarian, for example turning a chilli con carne into a chilli *non* carne. Ways to do this may be to use meat-mimicking products (for instance vegetarian mince) or by replacing the meat with legumes, pulses, beans, tofu, mushrooms, or jackfruit. All of these are great sources of protein and jackfruit and mushrooms are good at mimicking the texture of meat. If you are unsure what to replace the meat with, it can help to research vegetarian versions of the dish to see what other people have done. |
|  | 1. Use meat as a garnish or flavouring additive | Try to treat meat as a side to your meal, or even just a flavouring additive (such as a few bacon pieces in a pasta sauce). |
| **Try something new** | 1. Try a new vegetarian recipe | Try out a vegetarian or plant-based recipe you have never used before. If you’re looking for some extra inspiration, you can ask your friends or family what their favourite vegetarian or plant-based recipe is. Other good sources for vegetarian and plant-based recipes are: https://www.bbcgoodfood.com/recipes/category/vegetarian  https://www.vegsoc.org/recipes/ |
|  | 1. Try a new plant-based recipe | Try out a plant-based recipe you have never used before. If you’re looking for some extra inspiration, you can ask your friends or family what their favourite plant-based recipe is. Other good sources for plant-based recipes are:  https://cookingonabootstrap.com/category/vegan-recipes/  https://www.meatlessmonday.com/favorite-recipes/  https://www.bbcgoodfood.com/recipes/collection/vegan |
|  | 1. Try a meat-free alternative | Try a meat-mimicking product that you haven’t tried before. Most supermarkets now offer meat-free products which look and taste similar to the meat-based originals. For example, you can now get plant-based mince, sausages, burger patties or fillets. Many brands offer these products, so it’s best to do a bit of research first to find the best-rated option, or ask a friend for a recommendation. |
|  | 1. Go to a vegetarian or plant-based restaurant | Try out a vegetarian or plant-based restaurant in your area. If possible, order a new vegetarian or plant-based dish that you haven’t had before. To research vegetarian and vegan restaurants in your area, check out: https://www.happycow.net |
| **Cut out specific animal products** | 1. Eat no red meat | Eat no red meat today, including beef, lamb, pork, veal, or mutton. Red meat has the biggest environmental footprint, so reducing the consumption of this type of meat will have the largest impact. |
|  | 1. Eat no processed meat | Eat no processed meat today, including bacon, ham, sausages, salami, corned beef, and jerky. Processed meat is the type of meat that has been linked to the largest increase in risk to develop colorectal cancer and cardiovascular disease. |
|  | 1. Don’t cook or prepare any food with meat | When you cook your meals or prep your food today, make sure not to include any meat. |
|  | 1. Go vegetarian for the whole day | Go fully vegetarian for the day. That is, refrain from eating any foods that contain meat or fish. |
|  | 1. Go plant-based for the whole day | Go fully plant-based for the day. Make sure not to eat any animal-derived products, including meat, fish, dairy, and eggs. |
| **Limit your intake of animal products** | 1. Make at least one of your main meals vegetarian | Do not eat any meat or fish for at least one of your main meals today. |
|  | 1. Make your lunch and dinner vegetarian | Do not eat any meat or fish for lunch and dinner today. |
|  | 1. Order a vegetarian dish when eating out | When you eat out in a restaurant or get a take-away, make sure you order a dish that does not contain any meat or fish. |
|  | 1. Be an ‘at-home’ vegetarian | When you prepare food at home, refrain from using any meat or fish. |
|  | 1. Have only plant-based snacks | Make sure to have only plant-based snacks which do not contain any animal-derived products, including meat, fish, dairy products, and eggs. Great options are (dried) fruits, vegetables, nuts and seeds. If you are looking for a sweet treat, have a vegan-friendly biscuit, such as an Oreo. |
|  | 1. Have a plant-based lunch or dinner | Make sure that either your lunch or dinner contains no animal-derived products including meat, fish, dairy products, and eggs. |
| **Get family and friends involved** | 1. Organise a vegetarian party with friends | Invite your friends to a bring-and-share lunch or dinner party. Ask everyone to bring their favourite vegetarian dish and enjoy delicious food together. You might find some great ideas which you can steal for your next cooking session. |
|  | 1. Cook a vegetarian or plant-based meal for friends or family | Invite your family or friends to a home-cooked vegetarian or plant-based lunch or dinner. You can ask them what they thought of the meal – maybe you can come up with ways to spice up the recipe. |
