# Supplementary material for: Effectiveness of an Online Programme to Tackle Individual’s Meat Intake through SElf-regulation (OPTIMISE): A randomised controlled trial
Source: Eur J Nutr. 2022 Mar 4;61(5):2615–26. doi: 10.1007/s00394-022-02828-9 (PMC9279210; doi:10.1007/s00394-022-02828-9)
Supplement: Supplementary file 4 — Supplementary file4 (DOCX 301 KB) [file 394_2022_2828_MOESM4_ESM.docx]

**
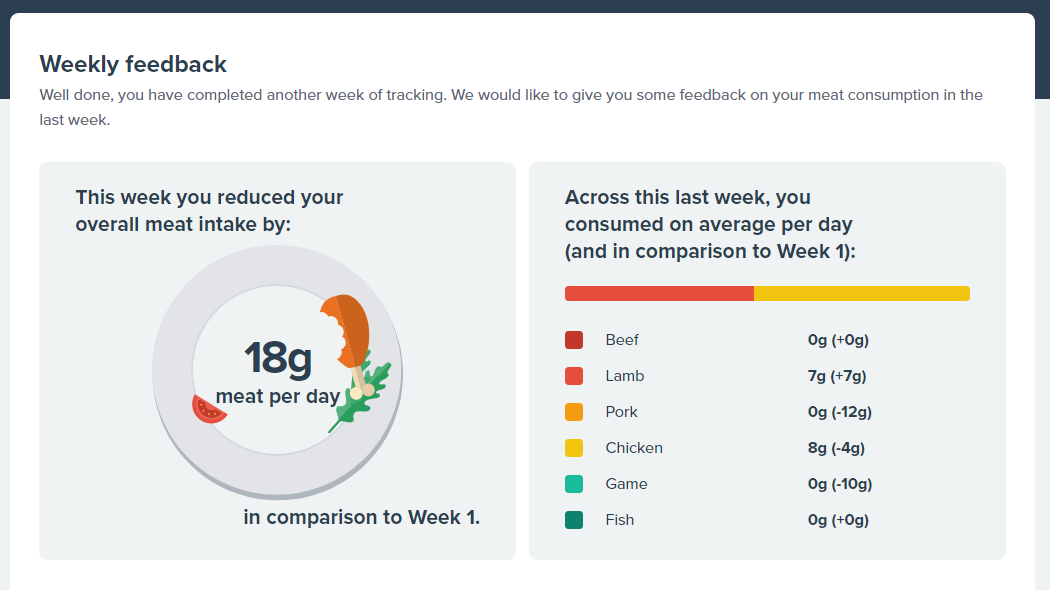
SI 4.** Example of weekly health and environmental feedback presented to participants provided during weeks 2-5.


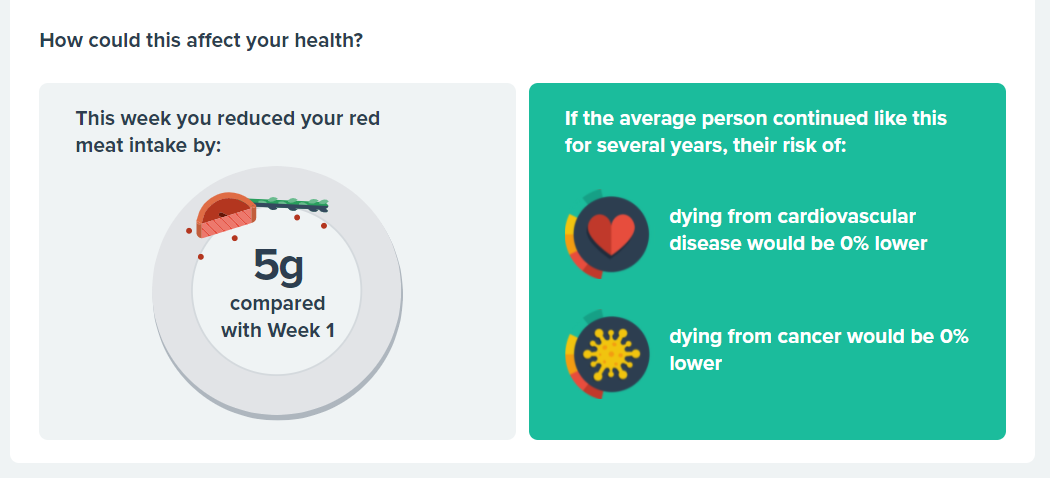
**
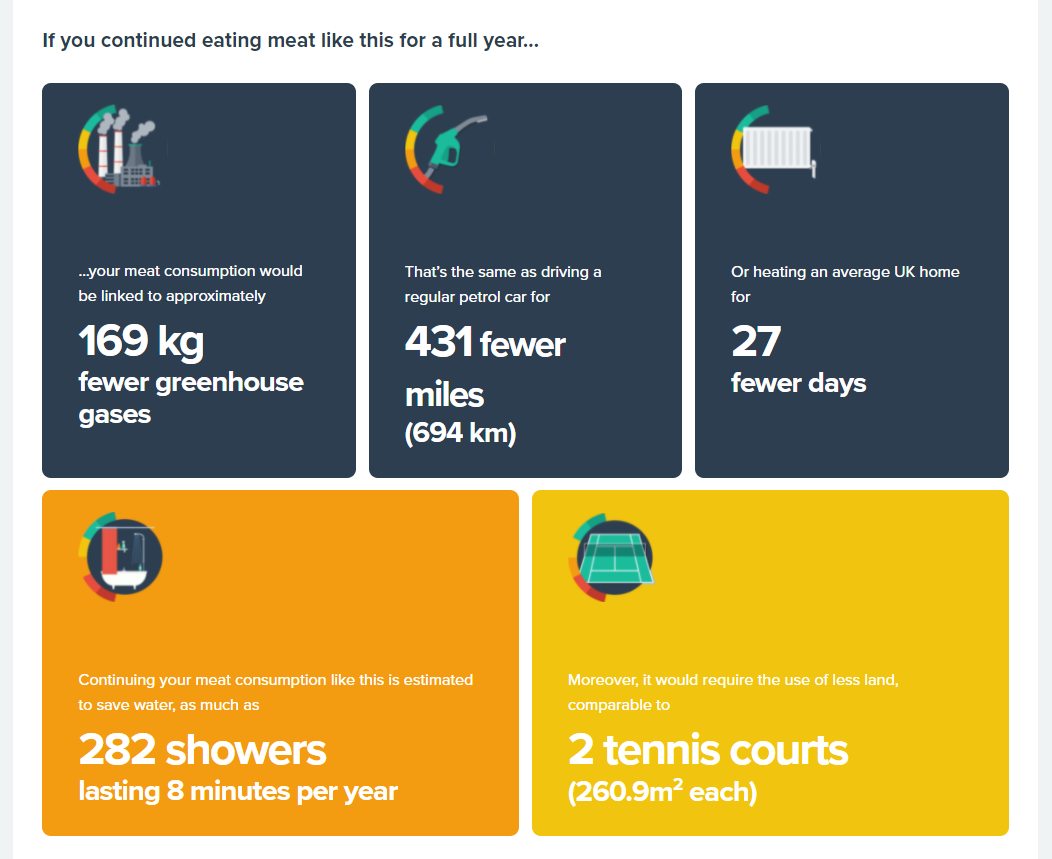
**
