# Supplementary material for: Effectiveness of an Online Programme to Tackle Individual’s Meat Intake through SElf-regulation (OPTIMISE): A randomised controlled trial
Source: Eur J Nutr. 2022 Mar 4;61(5):2615–26. doi: 10.1007/s00394-022-02828-9 (PMC9279210; doi:10.1007/s00394-022-02828-9)
Supplement: Supplementary file 5 — Supplementary file5 (DOCX 22 KB) [file 394_2022_2828_MOESM5_ESM.docx]

**SI 5.** The number of times each meat reduction action offered as part of OPTIMISE intervention was chosen during the active intervention phase (Weeks 2-5).
